# Bioluminescence Color-Tuning Firefly Luciferases: Engineering and Prospects for Real-Time Intracellular pH Imaging and Heavy Metal Biosensing

**DOI:** 10.3390/bios12060400

**Published:** 2022-06-10

**Authors:** Vadim R. Viviani, Gabriel F. Pelentir, Vanessa R. Bevilaqua

**Affiliations:** 1 Department of Physics, Chemistry and Mathematics, Federal University of São Carlos (UFSCar), Sorocaba 18052-780, Brazil; 2 Graduate Program of Biotechnology, Federal University of São Carlos (UFSCar), Sorocaba 18052-780, Brazil; gabrielpelentir@yahoo.com.br; 3 Faculty of Medical and Health Sciences, Pontifical Catholic University of São Paulo (PUC), Sorocaba 05014-901, Brazil; vani_bevilaqua8@hotmail.com

**Keywords:** bioluminescence, pH indication, heavy metals, cadmium, mercury, ratiometric biosensors, bioimaging

## Abstract

Firefly luciferases catalyze the efficient production of yellow-green light under normal physiological conditions, having been extensively used for bioanalytical purposes for over 5 decades. Under acidic conditions, high temperatures and the presence of heavy metals, they produce red light, a property that is called pH-sensitivity or pH-dependency. Despite the demand for physiological intracellular biosensors for pH and heavy metals, firefly luciferase pH and metal sensitivities were considered drawbacks in analytical assays. We first demonstrated that firefly luciferases and their pH and metal sensitivities can be harnessed to estimate intracellular pH variations and toxic metal concentrations through ratiometric analysis. Using *Macrolampis* sp2 firefly luciferase, the intracellular pH could be ratiometrically estimated in bacteria and then in mammalian cells. The luciferases of *Macrolampis* sp2 and *Cratomorphus distinctus* fireflies were also harnessed to ratiometrically estimate zinc, mercury and other toxic metal concentrations in the micromolar range. The temperature was also ratiometrically estimated using firefly luciferases. The identification and engineering of metal-binding sites have allowed the development of novel luciferases that are more specific to certain metals. The luciferase of the *Amydetes viviani* firefly was selected for its special sensitivity to cadmium and mercury, and for its stability at higher temperatures. These color-tuning luciferases can potentially be used with smartphones for hands-on field analysis of water contamination and biochemistry teaching assays. Thus, firefly luciferases are novel color-tuning sensors for intracellular pH and toxic metals. Furthermore, a single luciferase gene is potentially useful as a dual bioluminescent reporter to simultaneously report intracellular ATP and/or luciferase concentrations luminometrically, and pH or metal concentrations ratiometrically, providing a useful tool for real-time imaging of intracellular dynamics and stress.

## 1. Introduction

In the past decades, the firefly luciferin–luciferase system has been used in an enormous variety of bioanalytical applications. Firefly luciferase genes have been widely used as reporters in gene expression studies and for cell tracking in normal biological and pathological processes, including cell proliferation studies, cytotoxicity assays, and metastasis in model animals, emerging as a novel technology replacing animal testing by cell assays, and helping the pharmaceutical industry to find new therapeutic drugs [1,2,3]. Among these emerging technologies, firefly luciferases are being used as bioluminescent reporters to track pathogenic bacteria and viral infections, including SARS/COVID-19 [4], and in the development of high throughput drug screening assays (HTS) [5]. Firefly luciferases are also being used in luminescent biosensors [2,6].

Despite their many uses, luciferases have not been commonly used for intracellular pH indication and, with the exception of calcium, for metal biosensing. Currently, luminescent biosensors for pH and metals are mostly fluorescent. The use of bioluminescence in these types of bioluminescent sensors could potentially be useful for real-time bioimaging.

The estimation of intracellular pH is essential for cell homeostasis, cellular stress and intoxication. Intracellular and organelle pH variations are often associated with changes in the cellular cycle, such as cell division and apoptosis, and stress indicating pathologies such as inflammation, allergy and cancer [7,8]. On the other hand, metals may display either physiological functions, such as zinc and copper which are enzymatic cofactors, or toxic ones, especially the heavier metals such as mercury (Hg), cadmium (Cd) and lead (Pb). The estimated intracellular zinc concentration in human cells is around 200–300 µM [9]. Cadmium and mercury are among the most toxic metals causing serious damage to human health and the environment. The intracellular toxic concentrations of cadmium range from 1–300 µM [10], and its deleterious effects involve genotoxicity, generation of ROS, causing pathologies such as cancer, lung, bone and kidney injuries, and immunosuppression. Mercury’s toxic effects start from 1 to 100 µM, causing several cytotoxic effects. At 10 µM, HgCl_2_ causes three-fold more abnormalities/aberrations such as polycentrism of the chromosome and chromatid breakage [11]. In mammalian cells, 100 µM of HgCl_2_ affected cell division.

Several luminescent biosensors and indicators for pH were developed, most of them based on fluorescence intensity at a single wavelength [12,13,14,15], and some are based on fluorescence ratiometric analysis at distinct wavelengths [16]. Similarly, fluorescent biosensors for metals were also developed [17,18,19]. Bioluminescent sensors for metals relied first on bioluminescent bacterial *light off* sensors [20,21,22,23], and then on *light on* biosensors based on metal-inducible promoters coupled with luciferases [24].

A noteworthy but still overlooked property of firefly luciferases, from the bioanalytical point of view, is their pH and metal sensitivities, in which the bioluminescence color changes from green to red at an acidic pH, higher temperatures and in the presence of some heavy metals [25,26]. The mechanism of pH-sensitivity and bioluminescence color determination by firefly luciferases has been debated for over 5 decades, and recent studies by our group using firefly luciferases displaying distinct bioluminescence colors and spectral sensitivities [27,28,29,30] revealed the putative proton and metal binding sites responsible for pH and metal sensitivities [31].

Despite the demand for physiological intracellular biosensors for pH and heavy metals, firefly luciferase pH and metal sensitivities were originally considered drawbacks in analytical assays, and little attention was given to its potential utility for pH and metal indication. We recently demonstrated, for the first time, that firefly luciferases can be harnessed as ratiometric indicators of intracellular pH and heavy metals [32,33,34]. In this review, we briefly overview luminescent biosensors for pH and heavy metals and show the landmarks that lead to the exploration and engineering of firefly luciferases pH and metal sensitivities for ratiometric analysis of intracellular pH and toxic metals, providing their advantages, drawbacks and perspectives in real-time BL imaging.

## 2. An Overview of Current Luminescent Intracellular Sensors for pH and Heavy Metals

Luminescent biosensors for pH and metal detection can be divided into fluorescent and chemi- or bioluminescent. Most of the currently used luminescent biosensors are still fluorescent. Luminescent biosensors can be further divided into those based on the intensity at a single wavelength, in which the intensity increases or decreases in response to pH or metal concentration, and ratiometric ones, in which there are spectral changes that can be quantified by the ratio of intensities at different wavelengths. Each type of sensor has its own advantages and disadvantages. Herein, we briefly review the main types of fluorescent and bioluminescent sensors for pH and heavy metals.

**Fluorescent sensors.** The fluorescent sensors typically have the advantages of being simpler, convenient and inexpensive. Most of the currently used fluorescent biosensors are based on fluorescence intensity, which is linearly responsive to pH or increasing concentrations of metals, but may lack specificity [17]. On the other hand, the ratiometric fluorescent biosensors continue to increase, with advantages such as specificity and selectivity for metals. Whereas the fluorescence intensity at a single wavelength is sensitive to the actual concentration of the fluorophore and self-absorption variations, the ratio of intensities at different wavelengths is insensitive to fluorophore concentration, reflecting a more realistic concentration of the metal [17]. Despite their simplicity, fluorescent sensors have drawbacks such as the need for an external irradiation source, with auto-absorption by internal pigments, auto-fluorescence of the irradiated tissue, and phototoxicity effects caused by the irradiation with the external source.

**Fluorescent pH sensors.** For intracellular pH indication, specific fluorescent dyes and biosensors are usually used [7,8,12,13,14,15,17,35,36,37]. However, low molecular weight dyes may affect the physiology of the cell. Several pH-dependent fluorescent proteins (FPs) were also engineered and increasingly used to estimate intracellular pH [13,38,39,40], including ratiometric FPs based on either dual excitation or emission wavelengths [14,16,41,42,43]. However, as with any fluorescent dyes, FPs must be previously irradiated with blue light in order to emit fluorescence, which has potential drawbacks [12,44]. In order to reduce problems associated with autofluorescence and auto-absorption of light at shorter wavelengths in mammalian tissues, red-shifted emitting FPs have also been developed in the past 20 years [43]. Furthermore, because GFPs (Green Fluorescent Proteins) are quite stable proteins, they are usually well suited for ex vivo and microscopic fluorescence imaging, but not for real-time imaging.

**Fluorescent Metal sensors**. Fluorescent sensors were extensively used for distinct metals, either physiological ones such as calcium, zinc and copper, or toxic ones such as mercury, lead and cadmium [17]. Several fluorescent sensors to measure transition and heavy metals in biological systems use small fluorophores, whereas others use genetically encodable fluorescent proteins [40]. Here, we focus on luminescent sensors for physiological and toxic heavier metals such as zinc, copper, cadmium and mercury.

Ratiometric fluorescent sensors for the detection of Cu^2+^, in which increasing concentrations of Cu^2+^ decrease the ratio of fluorescence intensity at 470 nm and 355 nm (I_470_/I_355_), were developed [45]. Other ratiometric fluorescent sensors based on intramolecular charge transfer (ICT) using small fluorescent molecules with spectroscopic signatures of Zn^2+^, Hg^2+^and Pb^2+^ were also developed [46].

**Intracellular Zn^2+^**. Several fluorescent sensors that detect Zn^2+^ were developed [47,48,49,50], including ratiometric sensors [51] with detection limits lower than 50 µM. A highly selective fluorescent ratiometric sensor for Zn^2+^ based on bicarboxamidoquinoline, which uses the ratio of intensities at the wavelengths of 410 nm and 500 nm (I_500_/I_410_), was also developed [51]. Zinc fluorescent sensors based on fluorescent proteins are also popular [40]. Several zinc sensors are based on the principle of FRET, using zinc finger motifs in fluorescent proteins such as CFP (Cyan Fluorescent Protein) and YFP (Yellow Fluorescent Protein) [47,52].

**Hg^2+^ and Cd^2+^**. Fluorescent sensors for Hg^2+^ with sensitivities down to micromolar (µM) concentrations in water samples and living cells were also developed [50,53,54,55,56,57]. More recently, whole-cell fluorescent biosensors based on inducible *CadC* and *CadR* cadmium-binding proteins and GFP or Cherry were also developed, showing sensitivities to cadmium ranging from 0.1 to 400 µM [58]. Whole-cell *E.coli* biosensors based on *CadC* and GFP were also developed to detect cadmium in milk samples [59]. However, despite their sensitivity, a disadvantage of these biosensors is that the analysis requires longer times and considerable infrastructure because they require induction. Other fluorescent sensors include those based on FRET (Fluorescent Ressonance Energy Transfer) between ECFP (Cyan Fluorescent Protein) and *cpVenus* mediated by *CadR* cadmium-binding protein, with a Kd value around 250 nM [60]. Other biosensors based on ratiometric analysis of coumarin derivatives, with sensitivity between 40 and 660 pM [61], were also developed.

**Bioluminescent sensors**. Most bioluminescent biosensors are also based on luminescence intensity and are classified into *light off* and *light on* biosensors [6,62]. Non-specific *light off* biosensors based on natural and genetically transformed bioluminescent bacteria with the LUX operon [20,21,22,23,63,64] were the first whole-cell biosensors used to detect heavy metals and other toxic compounds. In the presence of these toxic metals, the respiratory chain of bacteria is inhibited and bioluminescence decreases. Bacterial *light off* biosensors using firefly luciferases that detect endogenous ATP were also proposed [21]. However, despite their practical use, the *light off* biosensors are rather non-specific regarding the toxic agents. On the other hand, *light on* bioluminescent biosensors based on the full *lux-CDABE*-operon were later developed to detect the bioavailability of Hg^2+^ based on the increase in the bioluminescence intensity [24,65,66]. A *light on* bacterial biosensor was also constructed using the firefly luciferase gene under the control of the *ars* promoter [65].

Bioluminescent reporters that use two or more emission colors are well known and were extensively used for dual and tricolor reporting in gene expression studies (www.promega.com.br/products/luciferase-assays/reporter-assays/dual_luciferase-reporter-assay-system/?catNum=E1910 (accessed 10 January 2022)) [67]. However, they usually rely on the use of two or more luciferase reporter genes [1] and are rather used to analyze gene expression instead of intracellular homeostasis indicators such as pH and metal concentrations.

The advantage of BL sensors over the fluorescent ones is usually the fact that they do not require external light irradiation in the UV and blue regions, eliminating problems associated with phototoxicity and autofluorescence. In general, they are more specific, emitting a specific light signal with a high signal-to-noise ratio that depends exclusively on the luciferin–luciferase reaction. Furthermore, luciferases, photoproteins and their luciferins usually do not display cytotoxicity. The main disadvantages, however, are the lower sensitivity of bioluminescence assays, due to much weaker signals than fluorescence, and their instability inside cells, which may reduce the potential signal. Despite that, their relative instability makes them advantageous for real-time imaging applications.

**Bioluminescent pH sensors**. Bioluminescence has not been commonly used to estimate pH. A photo-controllable bioluminescent protein based on firefly luciferase, which displays high sensitivity and low background, was constructed for real-time intracellular pH analysis [68]. **Calcium sensors**. The most widely known bioluminescent sensors for metals are aimed at detecting calcium, based on the well-known calcium-binding property of photoproteins and luciferases such as aequorin and obelin [69,70,71,72]. A bioluminescent sensor based on zinc finger protein was also developed [73]. **BRET sensors.** Bioluminescent ratiometric biosensors are much less common, and usually involve BRET (Bioluminescence Resonance Energy Transfer) systems, employing a luciferase or photoprotein and a fluorescent acceptor such as GFP which emit at different wavelengths [74]. Zhang et al. used *Renilla* RLuc8 as a donor for the permutated *Venus* FP acceptor to create the BRET-based tandem protein called pHlash, which shows an increase in emission ratio (525 nm/475 nm) as the pH increased [75,76]. Recently, Calflux, a BRET-based sensor for calcium that interposes a calcium-sensitive troponin-C sequence between the very bright Nanoluc and fluorescent proteins, was also constructed [77,78]. In neuronal cells, the release of calcium induces a conformational change of the troponin-C moiety, approximating NanoLuc and the fluorescent protein, allowing BRET to occur [79]. This ratiometric analysis provides a sensitive real-time estimation of released calcium with high a dynamic range, which is useful in conjunction with optogenetic probes such as melanopsin to measure calcium fluxes in neuronal cells. BRET sensors based on the fusion of NanoLuc and CFP (Cyan Fluorescent Protein) and eZincCh-2 were also developed to detect zinc, with sensitivities down to the pMolar range [80].

## 3. The Firefly Luciferases pH Sensitivity

**Figure 1:**
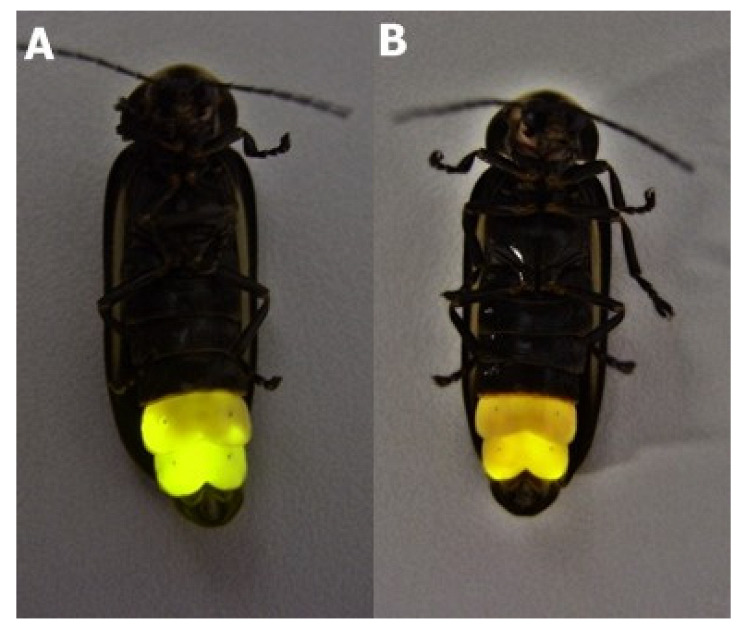
The *Macrolampis* sp2 firefly-induced bioluminescence display time-dependent color change after stimulation by anesthesia/adrenalin: (**A**) in the beginning just after adrenalin injection the color is yellow-green; (**B**) after 5 min, a subtle color change to yellow-orange is observable. Continuous glow stimulation may induce lantern acidification, causing the color change.

**Figure 2:**
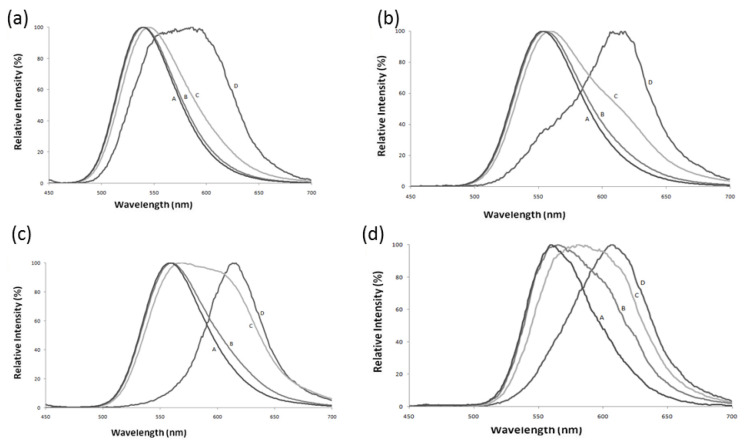
Effect of pH on bioluminescence spectra of four firefly luciferases displaying distinct pH sensitivities. Reprinted with permission from ref. [29]. Copyright 2011 Royal Society of Chemistry. (**a**) *Amydetes viviani*; (**b**) *Cratomorphus distinctus*; (**c**) *Photinus pyralis,* and (**d**) *Macrolampis* sp2. (A) pH 6.5; (B) pH 7.0; (C) pH 7.5, and (D) pH 8.0.

Firefly luciferases display a bioluminescence color varying from green to yellow-orange under normal physiological conditions or slightly alkaline pH [25,26]. However, when dying or stressed at higher temperatures, firefly bioluminescence color can change from the usual yellow-green to orange (Figure 1). At acidic pH, the light intensity decreases and the spectrum becomes red-shifted. A more detailed analysis showed that their bioluminescence spectra are composed of at least two spectral components, green and red emissions, but a third orange component was also considered [81]. Quantum yield measurements showed that at an alkaline pH, the green component predominates with a reported value of 41% for *P. pyralis* firefly, whereas at an acidic pH the green component decreases and the red component becomes predominant [81]. Distinct firefly luciferases were shown to display different degrees of pH sensitivity and proportions of green and red light (Figure 2) [29,82]. Usually, the most blue-shifted ones are also less sensitive to such factors [83,84]. Among the firefly luciferases we studied, the luciferase of *Amydetes viviani* is the most blue-shifted (539 nm) and also the least pH-sensitive, whereas those of *Cratomorphus distinctus* and *P. pyralis* display intermediate values and pH sensitivities, and that of *Macrolampis sp2* (569 nm) displays the broadest and more pH-sensitive spectrum [29].

**Figure 3:**
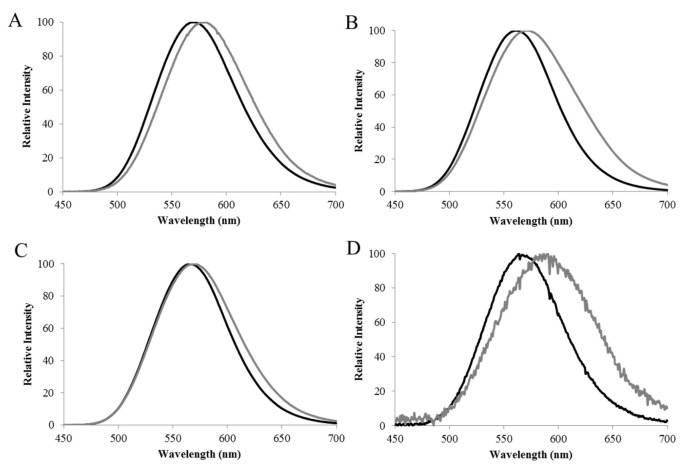
**The residue E354 is important for luciferase sensitivity to Zinc.** Effect of 2 mM zinc on the bioluminescence spectra of: (**A**) *Macrolampis sp2* wild-type; (**B**) *C. distinctus* wild-type; (**C**) *C. distinctus* E354N, and (**D**) *Macrolampis* sp2 N354E mutant (black line) without zinc, and (gray lines) in the presence of zinc. Reprinted with permission from ref. [33]. Copyright 2016 Springer.

**Metal sensitivity.** Almost all known adult lantern firefly luciferases also display bioluminescence spectra that are sensitive to certain heavier divalent metals such as zinc, nickel, mercury and lead. Similar to acidic pH, the presence of zinc was shown to decrease the emission quantum yield of the green component, leaving the red component predominant [77]. We found that firefly luciferases also varied in their degree of sensitivity to specific metals. For example, the luciferase of *Cratomorphus distinctus* displays a bioluminescence spectrum that is more sensitive to zinc, displaying a larger red shift in the presence of this metal (Figure 3) than *Macrolampis* sp2 firefly luciferase [33].

**Temperature sensitivity.** Temperature also affects the spectrum of firefly luciferases [25,26]. We demonstrated that luciferases with the most blue-shifted spectra display lower sensitivity to pH [83]. These observations corroborate the hypothesis that the active sites of the most blue-shifted luciferases are more rigid, whereas those of the most red-shifted and pH-sensitive luciferases are more flexible [85]. Mochizuki et al. also investigated the effect of temperature on the quantum yield of firefly luciferase [86]. Similar to the effect of pH and heavy metals, they showed that only the green component is temperature-sensitive, whereas the red and orange components are insensitive [86].

## 4. Identification of the pH-Sensor and Metal Binding Site of Firefly Luciferases

**Figure 4:**
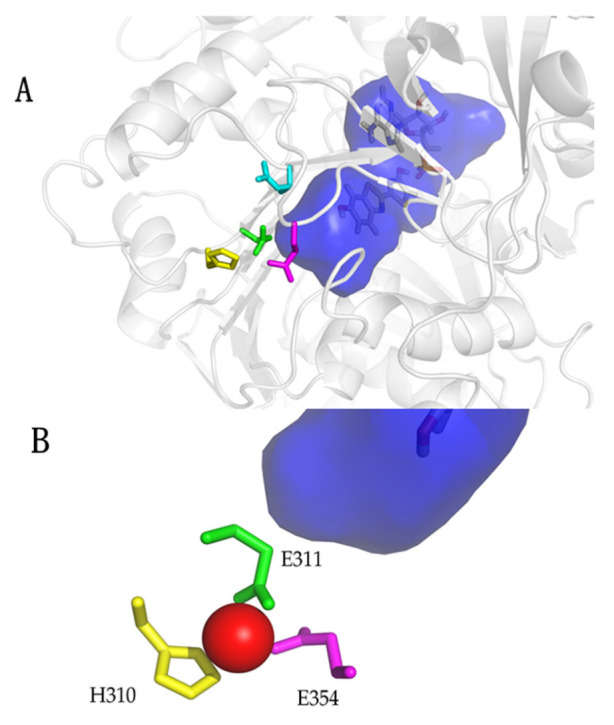
Structure of the metal-binding site in *Amydetes viviani* firefly luciferase: (**A**) Zoom showing the metal-binding site residues (yellow: H310; green: E311; magenta: E354) and oxyluciferin phenolate binding site (deep blue); (**B**) Metal-binding site showing zinc in red, being coordinated by the side chains of H310, E311 and E354. Reprinted from ref. [31].

**Figure 5:**
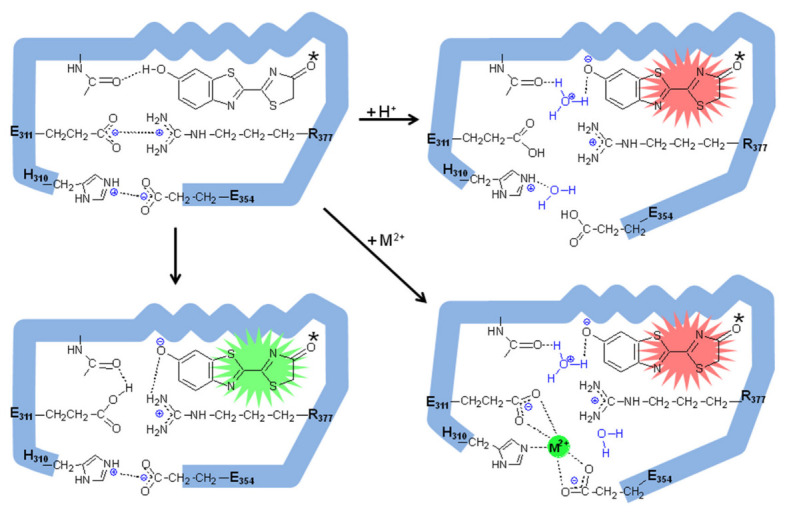
The proposed mechanism of pH and metal sensitivity in firefly luciferases involves the oxyluciferin phenol/phenolate group excited state proton transfer and electrostatic interactions between residues E311 and R337, and H310 and E354, which keep the active site closed. Whereas the keto form of excited oxyluciferin was considered the most likely emitter in this figure for simplicity, the process of keto-enol tautomerization in bioluminescence color determination can not be ruled out. Reprinted from ref. [31].

A comparison of the above and other pH-sensitive firefly luciferase primary structures, modeling studies and site-directed mutagenesis showed important substitutions that affected the proportion of green and red light, as well as the pH sensitivity [28,82,87]. Among them, the natural substitution E354N in *Macrolampis* firefly luciferase was clearly shown to be responsible for the broader and more red-shifted spectrum of this luciferase in relation to the closer *P.pyralis* and *Cratomorphus* luciferases [28]. Indeed, the substitution of the negatively charged E354 in *Cratomorphus* luciferase, by the neutral asparagine, which is naturally found in *Macrolampis* luciferase, decreased the sensitivity to zinc, also providing the first evidence of the importance of this site for metal binding [28]. The identification of the electrostatic pair H310 and E354 by modeling studies in firefly luciferase [82,88] also led to the identification of the close salt bridge E311 and R337, which later was shown to stabilize the closed hydrophobic conformation of the luciferase luciferin-binding site [89]. These results finally led to the identification of the pH-sensor and metal-binding site which involve the carboxylates of E311 and E354, and the imidazole or other nucleophilic side-chains of H310 (S/T310) as the proton and metal-binding sites (Figure 4) responsible for pH and metal sensitivities in firefly luciferases [31]. The identity and geometry of these sites, which include histidines and glutamates, indeed resemble the metal-binding sites of several zinc and cadmium metal-binding proteins [90,91]. The E311 carboxylate is a critical base responsible for pH sensitivity and is very likely to be involved in the chemiexcitation step by accepting the oxyluciferin phenolate released proton (Figure 5; [31]), whereas protons and metals such as zinc, lead and mercury, apparently bind to the basic or negatively charged side-chains of H310, E311 and E354, disrupting the active site salt bridges, polarizing the excited oxyluciferin phenolate binding site and promoting red light emission (Figure 5; [31]). Whereas the phenol group of excited oxyluciferin was shown to be critical for pH sensitivity and for bioluminescence color modulation [92], the influence of the keto-enol tautomerization may also exert a critical influence on bioluminescence color modulation [93].

## 5. Use of Firefly Luciferases as Color-Tuning Indicators of Intracellular pH

**Figure 6:**
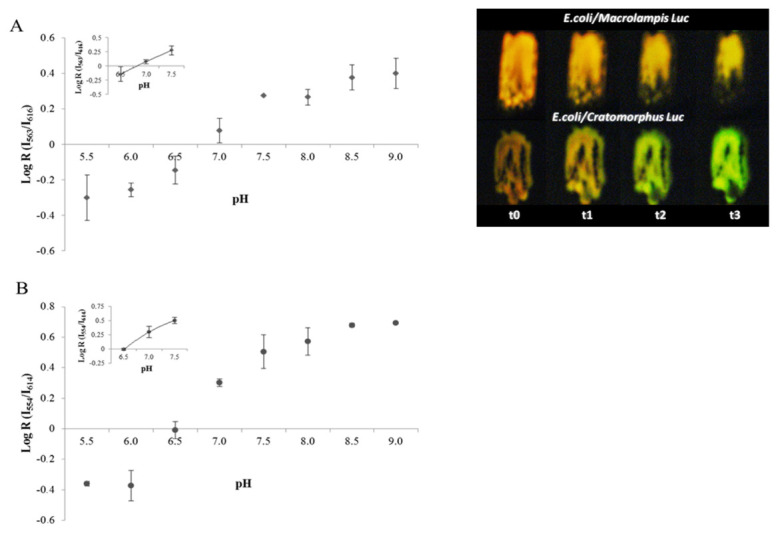
(**Left graph**) ratiometric analysis of pH in bacteria using: (**A**) *Macrolampis* and (**B**) *Cratomorphus* firefly luciferases; (**right image**) effect of pH on in vivo bioluminescence of *E. coli* colonies expressing *Macrolampis* and *Cratomorphus* firefly luciferases; (t_0_–t_3_) represent the time (minutes) when the bioluminescence image was taken after addition of acidic D-luciferin. Reprinted with permission from ref. [32]. Copyright 2014 Royal Society of Chemistry.

Considering that the firefly bioluminescence spectrum changes from green to red at an acidic pH, in 2005 we first proposed that pH sensitivity could be harnessed to estimate pH and metals [28]. We then analyzed the effect of the pH on the ratio of bioluminescence intensities in the green and red regions and found that there is a ratiometric relationship between pH 6.0 and 8.0 (Equation (1)) using *Macrolampis* sp2 and *Cratomorphus distinctus* firefly luciferases, allowing the estimation of intracellular pH changes in live *E. coli* bacteria [32]. We found that the bacterial intracellular pH was estimated to be approximately 7.1, in agreement with reported values using other methodologies. The results also showed that at an acidic pH, luciferin may act as a proton shuttle to the intracellular environment, initially acidifying the intracellular pH, producing reddish bioluminescence in bacteria (Figure 6), which then gradually changes to yellow-green, clearly showing that the intracellular buffering capacity of bacteria was recovered after some time.

(1) $$\mathrm{pH}=f\times R\left(\frac{I610}{I540}\right)$$

Equation (1) Ratiometric estimation of pH. *f* is the reason between pH and the ratio of intensities in the green and red regions in the linear range from pH 6.0 to 8.0.

**Figure 7:**
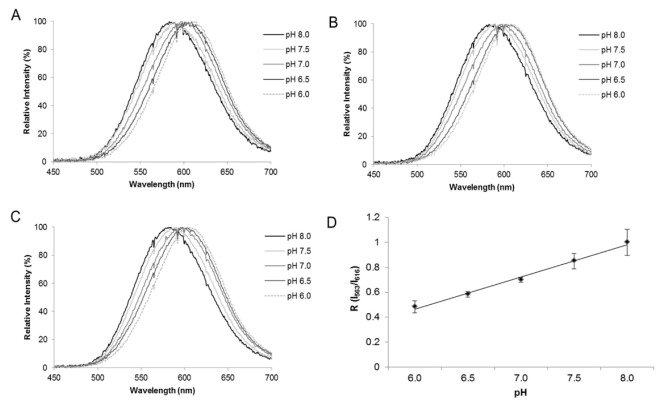
Bioluminescence spectra in COS-1 cells transfected with *Macrolampis* firefly luciferase carrying vector, pCMV-Mac, and ratiometric curves. Effect of pH in cells transfected with: (**A**) pCMV-Mac;cytoplasm; (**B**) pCMV-Mac-Nucleus; (**C**) pCMV-Mac with calibration buffer containing nigericin at different pHs (pH 6.0, 6.5, 7.0, 7.5 and 8.0); (**D**) ratiometric analysis between R (Igreen/Ired) and pH. Reprinted with permission from ref. [34]. Copyright 2019 Royal Society of Chemistry.

**Figure 8:**
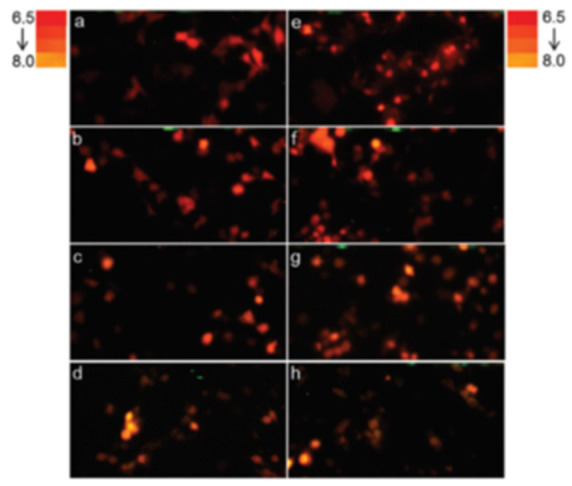
**Firefly luciferase color reporting of intracellular pH change in mammalian cells.** In vivo bioluminescence color change of COS-1 cells transfected with pCMV-Mac expressing *Macrolampis* firefly luciferase at different pH in calibration buffer containing nigericin. Reprinted with permission from ref. [34]. Copyright 2019 Royal Society of Chemistry. (left panels: **a**–**d**) luciferase directed to cytoplasm; (right panel: **e**–**h**) luciferase directed to nucleus; (**a**,**e**) pH 6.5; (**b**,**f**) pH 7.0; (**c**,**g**) pH 7.5; and (**d**,**h**) pH 8.0. The reddish bioluminescence indicates acidic pH, whereas the orange-yellow indicates more alkaline pH.

**pH indication at the single-cell level in Mammalian cells.** Using *Macrolampis* firefly luciferase, we also investigated whether this ratiometric methodology could be applied to estimate intracellular pH in mammalian cells [34]. We analyzed the intracellular pH in different cellular compartments, using filter-based luminometry, bioluminescence microscopy and spectroluminometry (Figure 7 and Figure 8), and found that the intracellular pH could be estimated, using Equation (1), at the single-cell level. The results also showed that the nucleus is more alkaline than the cytosol and that during cell division, the cytosol may also become more alkaline. However, the bioluminescence color of *Macrolampis* firefly luciferase is already quite red-shifted inside mammalian cells at 36 °C, which diminishes the visualization of color change.

## 6. Ratiometric Analysis of Temperature

**Figure 9:**
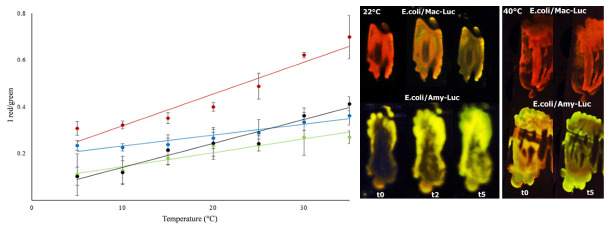
Comparison of the effect of temperature on the ratio of bioluminescence color of beetle luciferases: (**left graph**) ratiometric estimation of temperature using distinct firefly luciferases. (red) *Macrolampis* sp2; (black) *Photinus pyralis*; (green) *Cratomorphus distinctus,* and (blue) *Amydetes viviani*; (**right images**) in vivo bioluminescence color of *E.coli* colonies expressing *Macrolampis* sp2 (Mac-Luc) and *Amydetes viviani* (Amy-Luc) luciferases, after spraying D-luciferin, at 22 and 40 °C. Reprinted with permission from ref. [85]. Copyright 2014 Royal Society of Chemistry. One can see that the luciferases with steeper curves such as *Macrolampis* luciferase (red) are the most sensitive to temperature, and those with less steep curves, such as *Amydetes viviani* luciferase (blue), are the least sensitive.

We also showed a linear relationship between temperature and the ratio of green and red emissions (Figure 9; [85]), which could potentially be used to estimate intracellular temperature. However, it is questionable whether firefly luciferases can be practically used for temperature measurements.

**Compensation for temperature.** As shown previously, high temperatures also induce a red shift in the spectrum of firefly luciferases. Thus, at higher temperatures (37 °C), the spectrum of most firefly luciferases, such as that of the *Macrolampis* firefly, is already red-shifted, decreasing the magnitude of the observable color and spectral change at different pH values (Figure 9) and the potential resolution in bioimaging analysis. Therefore, for bioanalytical assays performed at higher temperatures, it is important to compensate for the temperature effect. The ratiometric curves of temperature for a specific luciferase could in principle potentially be used to compensate for the spectral changes caused by pH at higher temperatures. Alternatively, luciferases such as the *Amydetes* firefly, which are less sensitive to temperature in the ratiometric analysis of pH, could be used.

## 7. Use of Firefly Luciferases as Color-Tuning Sensors for Heavy Metals

**Figure 10:**
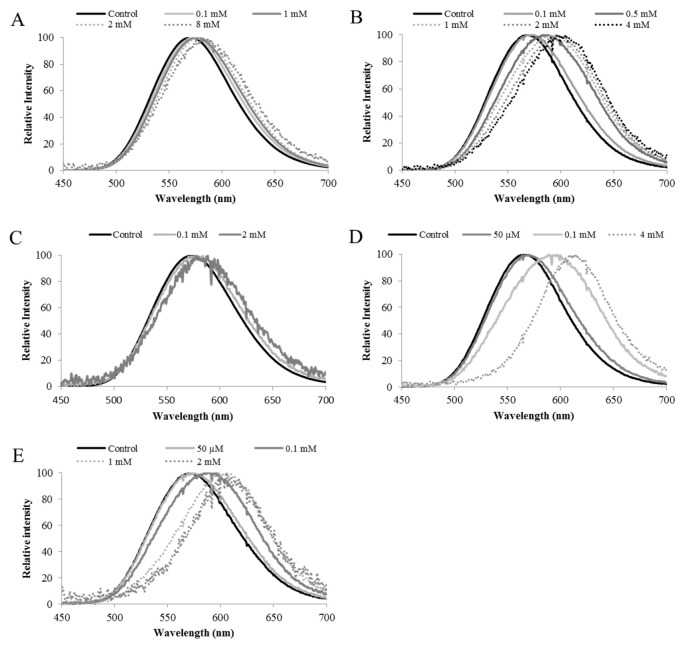
Bioluminescence spectra of *Macrolampis* sp2 firefly luciferase and its mutants showing spectral change at different concentrations of ZnSO_4_: (**A**) Wild-type; (**B**) N354H; (**C**) H310C; (**D**) N354C; (**E**) H310C/N315C; Reprinted with permission from ref. [33]. Copyright 2016 Springer.

Based on a similar principle of pH sensitivity, we then analyzed the effect of different heavy metal concentrations on the ratio of green and red bioluminescence intensities using *Macrolampis* and *Cratomorphus* firefly luciferases and found that there is also a linear relationship (Figure 10; [33]) in the range of ~15–2000 μM, depending on the metal. The metal concentration can be estimated by the product of the ratio (R) of the bioluminescence intensity in red and green, according to Equation (2).

(2) $$\left[Me^{2+}\right]=f\cdot R\left(\frac{I610}{I540}\right)$$

Equation (2) Ratiometric estimation of heavy metal concentration. *f* is the ratio between metal concentration and the ratio of intensities in the red and green regions.

Different firefly luciferases display distinct natural degrees of sensitivity to metals such as zinc, copper, cadmium and mercury. The luciferase of *Cratomorphus* and *Photinus pyralis*, for example, are more sensitive to zinc than *Macrolampis* firefly luciferase (Figure 3; Table 1).

## 8. Selection of Metal-Sensitive Luciferases and Engineering of the Metal-Binding Site

Based on the identified structure of the metal-binding site in firefly luciferases, and predictors of metal-binding sites in other proteins [91,94,95], we already started to engineer the metal-binding site of firefly luciferases by mutating the residues H310 and E/N354, in order to change the metal sensitivity. Furthermore, considering the natural differences in metal sensitivity displayed by some firefly luciferases, we started to select luciferases better suited for specific metals. For example, *Cratomorphus distinctus* larval firefly luciferase is more sensitive to zinc (Figure 3), whereas *Macrolampis sp2* firefly luciferase is more sensitive to mercury. Table 1 compares the spectral sensitivity and other bioluminescence properties and applications of firefly luciferases and their mutants.

Indeed, by changing H310 and E354, we could change the metal sensitivity of the *Macrolampis* firefly luciferase (Figure 10), as can be seen by the detection limits that cause a minimal spectral change and the spectral amplitude caused by the metal (Table 1; [33]). As expected by the chelating property of the side-chains of the substituted residues, the substitution of N354 by His increased the sensitivity to nickel but also for mercury, whereas the substitution of H310 by cysteine increased the sensitivity for zinc (detection limit 20 µM) and mercury (detection limit 15 µM). The double mutant H310C/N354C showed a considerably increased sensitivity to zinc (detection limit 15 µM).

**Figure 11:**
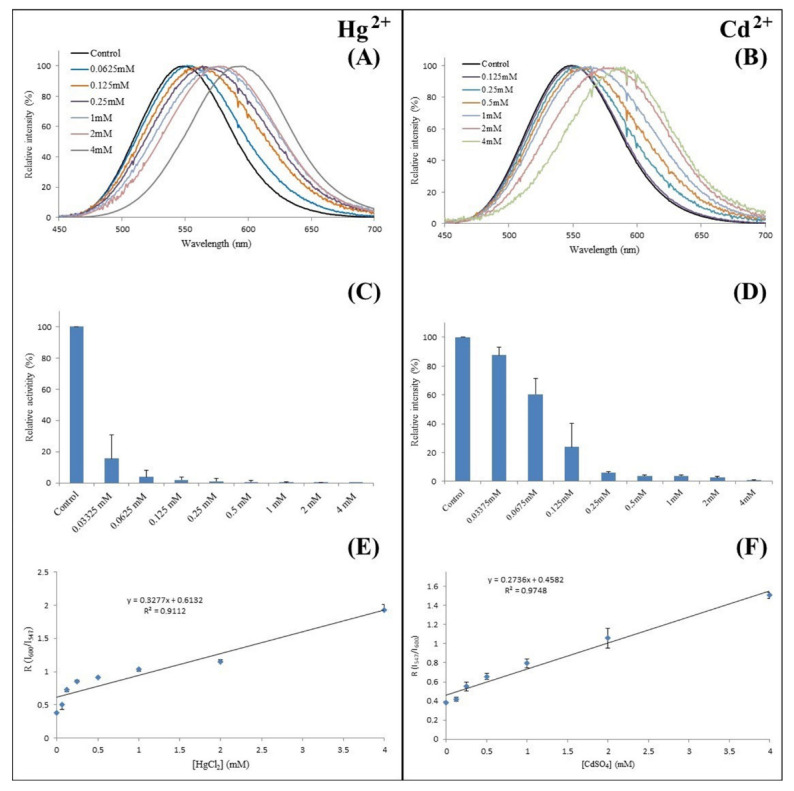
Use of *Amydetes viviani* firefly luciferase as cadmium- and mercury-selective luciferase: (**A**,**B**) bioluminescence spectra in presence of mercury and cadmium; (**C**,**D**) bioluminescence activity in presence of mercury and cadmium; (**E**,**F**) ratio of luminescence intensities in green and red regions in presence of mercury and cadmium. Reprinted with permission from ref. [84]. Copyright 2019 National Library of Medicine.

**A cadmium- and mercury-selective luciferase.** More recently, we found that *Amydetes viviani* firefly luciferase, which is the most blue-shifted and least pH-sensitive among the studied adult firefly luciferases [29], displays spectral sensitivity exclusively to Cd and Hg at concentrations below 2 mM (Figure 11) [84], in contrast to other studied firefly luciferases studied, which display similar spectral sensitivities to different metals such as zinc, cadmium and mercury at the same concentration. The minimum cadmium concentration that caused a measurable red shift in *Amydetes* luciferase was estimated to be 100 μM, whereas that for mercury was 62 μM. Furthermore, the activity of cadmium was less affected than that of mercury (Figure 11). Such spectral sensitivity and the improved catalytic properties make *Amydetes* firefly luciferase particularly suitable as an enzymatic sensor for cadmium detection.

## 9. Smartphone Detection of Cadmium Contamination in Water

**Figure 12:**
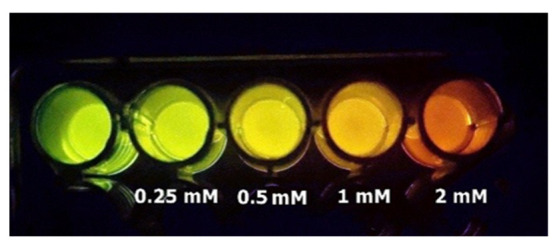
Smartphone detection of bioluminescence color tuning by cadmium, using *Amydetes viviani* firefly luciferase.

We are harnessing *Amydetes* luciferase to detect cadmium and mercury contamination in water samples using smartphones (Figure 12). A contaminated water sample can be concentrated and then assayed with the *Amydetes* luciferase assay solution. If the water sample contained between 0.1–1 µM of cadmium or mercury salts, the bioluminescence color change could be visually observed and photographed using a standard CCD camera-based smartphone. This methodology provides a potentially easy, affordable and *hands-on* bioluminescent biosensor for fieldwork, and is very useful for teaching laboratory classes on the effects of heavy metals on protein function in biochemistry courses.

## 10. Is it Possible to Report in Two Dimensions?

Dual reporting usually involves the use of two or more luciferase genes that emit distinct BL colors. Such a methodology is generally used to investigate gene expression processes in cells. We showed that firefly luciferases can be used as sensitive tools for the ratiometric analysis of intracellular pH or water contamination by toxic metals. The main advantage of this approach is the use of a single luciferase gene that does not require other fluorescent acceptors such as in BRET or other accessory proteins because the luciferase itself is the proteic sensor, making the analysis simpler, potentially cost-effective and free from interference.

Because luciferase bioluminescent activity has already been used to quantify intracellular ATP or gene expression, one may have the double advantage of using a single luciferase gene to report in two dimensions, the luminescence intensity to report intracellular ATP concentration or luciferase expression and the spectral ratiometric analysis to report a specific homeostatic event such as intracellular pH changes, variations in free intracellular concentrations of physiologic metals, or the presence of toxic metals. Such an approach could be a very powerful tool for the real-time assessment of cellular stress and intoxication in cell assays.

However, to be more effective and to gain more information, it is important to assess ATP concentrations or luciferase expression separately. Therefore, below, we briefly analyzed the gene expression and ATP levels independently.

**Gene expression.** Luciferases are widely used as reporter genes. Bioluminescence can be used to determine the cellular location, control and level of gene expression. At saturating concentrations of substrates, which usually occur inside the cells, the intensity of bioluminescence (*I*) depends exclusively on the concentration of expressed luciferase [*Luc*], its catalytic constant (*k*_cat_) and the quantum yield (*QY*) (Equation (3)). Once the quantum yield (*QY*) and *k*_cat_ of some firefly luciferases are known [96], it is in principle possible to estimate the intracellular luciferase concentration at saturating substrate concentrations, according to Equation (3).

(3) I=QY×kcat×[Luc]

Equation (3). Equation showing the relationship between bioluminescence intensity (I) and luciferase concentration [Luc]. *k*_cat_ is the catalytic constant (ratio of I/[luciferase]) and QY is the quantum yield of the bioluminescence reaction (number of emitted photons by number of luciferin molecules oxidized).

Recently, the bioluminescence intensity emitted by a single mammalian cell-expressing beetle luciferase was estimated to be between 1100–2500 photons s^−1^, and the number of luciferase molecules expressed inside the cell was estimated to be approximately 2000–3000 molecules [97]. However, one limiting factor for attaining high intensity could be oxygen availability, especially in deep tissues under anoxic conditions.

**ATP assays.** Reporting ATP inside cells using firefly luciferase is also possible in principle. However, the fluctuation of luciferase expression inside the cells may challenge the precise (ATP) estimation. Koop and Cobbold (1993) could first successfully measure intracellular ATP in cardiomyocytes and hepatocytes by microinjection of firefly luciferase. They showed that upon treating cells with the glycolysis and respiratory chain inhibitors, deoxyglucose and cyanide, a drop of bioluminescence signal usually took a long time (20–75 min), which could be attributed to the saturation of luciferase with the intracellular ATP and with the side-production of ATP by β-oxidation [98].

The K_M_ for ATP is well known for most luciferases, usually ranging from 20–250 μM. The expected concentration of ATP in healthy cells usually ranges from 2–8 mM, which obviously saturates most luciferases. Considering that the usual concentrations of ATP in a healthy cell are well above the reported K_M_ values for ATP for most beetle luciferases, it would be possible to estimate decreases in ATP concentrations only below 200 μM, which is of little practical use, given that most cells would die at such low concentrations. An alternative to estimating physiological intracellular ATP fluctuations is the use of luciferases with higher K_M_ values for ATP, such as those of some railroad worms which are, however, pH-insensitive [99,100]. Furthermore, estimating ATP concentrations by luminescence intensity, simultaneously with pH changes by ratiometric analysis, poses additional difficulties because it is well known that the quantum yield and emission intensity also decrease at low pH or in the presence of heavy metals. However, considering that the K_M_ value for ATP can be estimated at different pH values, it is in principle also possible to estimate the K_M_ at different pH and wavelengths to compensate for the intensity changes.

In summary, intracellular luciferase concentrations could be estimated if the quantum yield of the luciferase and equipment intensity factor are provided. On the other hand, intracellular ATP concentrations could be estimated, providing a luciferase with a high enough K_M_ value (>250 µM), and a curve of the effect of pH on K_M_ values. If ATP or luciferase intracellular concentrations could be estimated, then the use of a single firefly luciferase gene could be very useful as a dual reporter, providing a holistic image of cell homeostasis during normal biological processes such as cell division, apoptosis and fermentation, or pathological processes such as inflammation, allergy, cancer and cell toxicity assays in the pharmaceutical and cosmetic industries.

## 11. Drawbacks and Perspectives: Comparison with Other Luminescent Biosensors

The color-tuning firefly luciferases described here provide a novel kind of ratiometric luminescent sensor for intracellular pH and toxic metals, with the main advantage of using a single firefly luciferase. The main drawbacks are the lower sensitivity of the assay in relation to fluorescent sensors, and the lower specificity, since these luciferases respond to either pH or some heavy metals. However, the signal-to-noise ratio is in general much better for bioluminescent sources, which have no competing intracellular chemiluminescent reactions. The fluorescent sensors, despite being more sensitive and simpler, have drawbacks such as the need for external irradiation by a light source to excite fluorescence, problems associated with phototoxicity due to irradiation in the ultraviolet and blue regions, self-absorption of the irradiated light and autofluorescence of endogenous compounds, which decrease the signal-to-noise ratio. Similar to the fluorescent ratiometric biosensors, the firefly luciferase bioluminescent ratiometric biosensors described here also eliminate problems associated with changes in luciferase expression and its inactivation, which are usually drawbacks in luminescent biosensors based on the light intensity at a single wavelength. Furthermore, because luciferases are unstable proteins, they do not accumulate inside cells as GFP does. The lower luminescent signal produced by the shorter half-life of luciferase could be overcome by the use of brighter luciferases, such as that of *Amydetes viviani* firefly. Altogether, these properties make pH-sensitive firefly luciferases promising bioindicators for real-time bioimaging and measurement of intracellular pH changes.

**pH range.** The pH sensitivity of firefly luciferases between pH 6 and 8.5 is well suited for physiological analysis but can not be used for analysis beyond such pH values. The distinct intensities produced at different pH values could also be a concern in the use of firefly luciferase in such analysis when using equipment based on photomultipliers that display lower sensitivities in the red region. However, such problems can be circumvented by using CCD-provided equipment which show more linear spectral photoresponse.

**Tissue absorption**. Another disadvantage of using firefly luciferases as pH indicators is their use for bioimaging purposes in deep live mammalian animals because firefly luciferases emit light in the range of green-orange/red, which is considerably absorbed by hemoglobin and other pigments. Therefore, currently, firefly luciferases can only be used to report pH in cell cultures and superficial tissues. This problem could be circumvented by engineering more far red-shifted luciferases, which are pH-sensitive with pH-sensitive luciferin analogs. It was shown that some luciferin analogs that preserve the phenol hydroxyl group also preserve pH sensitivity emitting in the FR and NIR [101].

**Working at high temperatures.** As shown, firefly luciferases display red-shifted spectra at higher temperatures, which may decrease the sensitivity of the pH estimation, especially in bioimaging. Luciferases can be engineered to improve the stability of bioluminescence spectra at higher temperatures. However, there is a *trade-off* between pH and temperature sensitivity: decreasing temperature sensitivity may also decrease pH sensitivity. The luciferase of *Amydetes* firefly is thermally more stable while preserving pH and metal sensitivity. We believe that further research is required to improve these properties.

**Metal sensitivity and selectivity.** The lower selectivity and sensitivity of ratiometric assays for metals using pH-sensitive firefly luciferases when compared to other reported fluorescent sensors are major drawbacks. As shown, the sensitivity and selectivity for specific metals can be improved by engineering the metal-binding site. Among the firefly luciferases we investigated (Table 1), the *Macrolampis* luciferase mutant N354C was the most sensitive to mercury, with a detection limit of 15 µM, however, it was not specific enough, displaying sensitivity to other metals, mainly zinc. The mutant H310C/N354C was the most sensitive to zinc with a detection limit of 15 µM, a value that is close to some fluorescent intracellular biosensors, and a spectral amplitude of 38 nm. However, similarly to the N354C mutant, the H310C/N354C also lacked selectivity, displaying sensitivity to other metals. Overall, currently, the *Amydetes* firefly luciferase was the one that displayed the best combination of properties, with special selectivity to cadmium and mercury, with detection limits of 100 µM for cadmium and 60 µM for mercury, and a spectral amplitude above 38 nm. For detecting heavy metal contamination in water samples, the lower sensitivity can be circumvented by concentrating the water samples to a volume in which the concentration of the metal reaches the micromolar (µM) range, as we have shown. However, for intracellular biosensing, the luciferase sensors are not as sensitive yet as the fluorescent sensors, which display sensitivity in the upper nanomolar to micromolar range. We anticipate that there may be some space for further improvement of this sensitivity. Nevertheless, although firefly luciferases are not specific metal-binding proteins, they could just be used as average intracellular sensing proteins to estimate toxic metal concentrations that cause average enzymatic and metabolic inhibition. Finally, considering that, often, the effect of these toxic metals is additive, luciferases could be used as fast average enzymatic indicators of total heavy metal concentration in a water sample.

## 12. Conclusions

Firefly luciferases can be harnessed to ratiometrically estimate intracellular pH in living bacteria and mammalian cells in the range from pH 6.0 to 8.5, and to estimate the concentration of physiologic metals such as Zn, and toxic heavy metals such as Hg, Cd and Pb down to the μM range. Whereas some firefly luciferases are not selective for distinct heavy metals, we showed that new luciferases can be selected or engineered in their metal-binding sites, providing a palette of selective metal color-tuning luciferases. Although firefly luciferases are not yet sensitive as intracellular sensors of these toxic metals, they could nevertheless be used in field enzymatic bioluminescent sensors of specific toxic metals and overall mixtures, and for educational purposes showing the average effect of these metals on protein function. The use of firefly luciferases as ratiometric biosensors for pH offers several advantages over other luminescent reporter genes: (1) the sensitivity of the ratiometric analysis, which is devoid of problems associated with fluorescence, such as self-absorption, autofluorescence and phototoxicity; (2) the ratiometric analysis is not affected by the level of luciferase expression or substrate concentrations, which could be a problem when using fluorescent and bioluminescent reporter genes based on intensity; (3) the relative instability of firefly luciferases, which is more appropriate for real-time in vivo imaging, and (4) and the possibility of using a single bioluminescent reporter gene for simultaneous dual reporting of gene expression or ATP concentration inside the cells using the light intensity, and intracellular pH changes using color tuning or the spectral ratiometric analysis. These color-tuning luciferases, together with the development of more sensitive detection devices such as CCD cameras, BL microscopes and filter luminometers, may provide a novel and promising technology to probe intracellular stress in cell assays. Furthermore, the development and miniaturization of photodetection systems, such as smartphones with sensitive CCD cameras, may enable the development of *hands-on* biosensors for *in loco* cell toxicity assays, and for field estimation of bioavailability and water contamination by toxic metals.

## Figures and Tables

**Table 1:** pH-sensing and metal binding properties of firefly luciferasesand their engineered forms, and potential applicability.

Luciferase	RA (%)	pH Sensitivity (nm)	RA ZnSO_4_ 1 mM (%)	ZnSO_4_ Detection Limit * (µM)	ZnSO_4_ Spectral Shift (nm) **	RA CdSO_4_ 1 mM (%)	CdSO_4_ Detection Limit * (µM)	CdSO_4_ Spectral Shift (nm) **	RA HgCl_2_ 1 mM (%)	HgCl_2_ Detection Limit * (µM)	HgCl_2_ Spectral Shift (nm) **	Applicability
***Macrolampis* sp2**	100	569–616	77	500	9	36	890	19	18	170	34	**pH-indicator**
N354H	5.7	568–615	20	110	30	21	2000	5	7	150	37	**pH-indicator**
N354C	75	564–606	8	20	47	13	15	50	6	15	37	**Zinc, Cadmium and Mercury enzymatic sensor**
H310C	62	573–613	20	100	15	-	3500	0	11	90	34	**pH-indicator**
H310C/N354	-	-	15	15	34	11	260	41	4	30	36	**Zinc and Mercury enzymatic sensors**
N354E	-	-	90	225	23	14	4000	0	7	130	21	
***Cratomorphus distinctus***	100	554–614	-	-	36	-	-	-	-	-	46	**pH-indicator**
***Amydetes viviani***	100	549–596	2	2000	4	4	100	29	1,5	60	33	**Cadmium and Mercury enzymatic sensors; thermostable pH indicator**

* The detection limit is the concentration of a given metal which red-shifts the spectrum by no less than 5 nm. ** Maximum spectral shift observed at the metal concentration of 2 mM.

## Data Availability

Not applicable.
